# Effect of Mold Opening Process on Microporous Structure and Properties of Microcellular Polylactide–Polylactide Nanocomposites

**DOI:** 10.3390/polym10050554

**Published:** 2018-05-21

**Authors:** Pengcheng Xie, Gaojian Wu, Zhida Cao, Zhizhong Han, Youchen Zhang, Ying An, Weimin Yang

**Affiliations:** 1 College of Mechanical and Electrical Engineering, Beijing University of Chemical Technology, Beijing 100029, China; xiepengcheng@gmail.com (P.X.); wu039879202@163.com (G.W.); 18811414263@163.com (Z.C.); zxy1124895574@126.com (Z.H.); zhangyc@mail.buct.edu.cn (Y.Z.); anying@mail.buct.edu.cn (Y.A.); 2 State Key Laboratory of Organic-Inorganic Composites, Beijing University of Chemical Technology, Beijing 100029, China

**Keywords:** microcellular PLA, mold opening, pressure drop rates, cell structure, mechanical properties

## Abstract

Cell structure is a key factor that determines the final properties of microcellular polylactide (PLA) product. In the mold opening process, adjusting the rate of mold opening can effectively control cell structure. PLA and PLA composites with a void fraction as high as 50% were fabricated using the mold opening technique. The effects of mold opening rate and the addition of nanoclay on the cell structure, mechanical properties, and surface quality of microcellular PLA and PLA composites samples were investigated. The results showed that finer cell structure was received in the microcellular PLA samples and the surface quality was improved effectively when decreasing the rate of mold opening. The effect of mold opening rate on the foaming behavior of microcellular PLA–nanoclay was the same as that of microcellular PLA. The addition of 5 wt % nanoclay significantly improved the foaming properties, such as cell density, cell size, and structural uniformity, which consequently enhanced the mechanical properties of foams and the surface quality.

## 1. Introduction

Polylactide (PLA) is biodegradable, which makes it be a good alternative of the currently used polymer products to solve increasingly serious environmental problems [1,2]. Microcellular PLA has many advantages, such as low carbon and high specific strength. Nowadays, it has been widely applied in consumer goods and biological, transportation, and architecture parts [3,4].

In microcellular products, the structural parameters of the micro pores determine the performance of the material [5], such as cell diameter and density. For PLA products, the diameter and density of the cell was suggested to be about 10 μm and 10^9^ cells/cm^3^, respectively [6]. Controlling the microporous structure is an important method to optimize the performance of the microcellular PLA products [7,8]. Low-pressure microcellular injection is widely used to enhance porosity and control micro construction [9]. Microcellular PLA can get an average cell diameter of as large as 20 μm and a porosity of over 30% [10]. However, it is still difficult to control the growth and coalescence of cells during the micro-foam process, because there is shear stress during the filling stage [11]. High-pressure microcellular injection molding, named MuCell technology, can control cells well, and it is uncomplicated to control cell nucleation, growth, and coalescence [12,13]. With this technology, the diameter of micro cells can range from 3 μm to 40 μm [14]. Compared with low-pressure microcellular injection molding, obtaining higher porosity is a challenge for high-pressure FIM (foam injection molding), and it is usually less than 15%, though a very uniform foam structure can be obtained [15].

In order to obtain finer cells and higher porosity with high-pressure FIM, controlling mold opening (MO) is more practical to change the pressure and mold cavity, which are the key factors that determine the cell quality and density [16,17,18]. By this way, cell quality would be improved while getting a fine overall performance [17]. In this technology, polymer–gas mixture is first fully injected into the mold cavity with high pressure. Then, the mold or core insert expands in a certain distance and a great number of bubbles arise in the polymer melt when the pressure drops down [18]. Some researchers changed the in-mold size to improve the porosity of PP and PET by 70% [19]. Moreover, Sporrer and Wong et al. studied the injection parameters of MO foaming to confine micro structure and to improve the overall quality [20,21]. Sadik et al. investigated the skin, core, and overall mechanical properties of polymeric sandwich foams in comparison with the predictive models [22]. Earlier, Ishikawa et al. studied fast MO to get higher porosity [16]. Michaeli et al. controlled the cell morphologies and densities of foamed samples by adjusting the opening distance, and analyzed the failure behavior of foams [23]. Valentina explored the filling and cooling stages by Moldflow simulations, and compared the morphological, rheological, and mechanical properties of two different PLA grades by foam injection molding with mold opening process [24]. Ruiz et al. analyzed the morphology of microcellular injected polypropylene using chemical blowing agents and a combination of core-back and GCP processes [25]. With regard to the cell-nucleation mechanisms in high-pressure foam injection molding, Shaayegan et al. studied the mechanism of controlled bubble nucleation in high pressure foam injection molding via in situ visualization using polystyrene and supercritical carbon dioxide system. It was observed that the rate of bubble nucleation is a power function of the cavity pressure which determines the final cell structure of the foam molded part [26]. In order to obtain a high pressure drop rate, they applied a melt-packing pressure before mold opening and decreased melt compression, which increased the cell density and enhanced the structural uniformity [27]. Ameli et al. investigated the foaming behaviors and mechanical properties of the PLA foams with mold opening technique and reported that mold opening and the addition of both talc and nanoclay enhanced the PLA foams’ structure with a finer and more uniform cell structure (cell size <50 μm) at a void fraction of 55% [28]. However, little work has been systematically done to understand the effects of higher mold opening rate on the cell-nucleation mechanism and the surface quality of microcellular PLA.

In this study, the mold opening process was adopted to fabricate microcellular PLA and PLA composite with void fractions as high as 50%. We explored the effect of mold opening rate on the cell structure, mechanical properties, and surface quality of the product. Two different foamed material systems were investigated with four pressure dropping rates: pure PLA and PLA with 5 wt % nanoclay (PLA–nanoclay). The effect of the addition of nanoclay on these properties of PLA–nanoclay composition was also identified.

## 2. Experimental Procedure

### 2.1. Materials

The polymer used in this study was the commercially available PLA, Ingeo^TM^ 2002D (NatureWorks LLC, Minnetonka, MN, USA). The PLA was in pellet form and had a melt flow index of about 11.2 g/10 min. (210 °C/2.16 kg) with a density of 1.24 g/cm^3^ and a D-lactide molar content of 4.1%. The nucleating agent, nanoclay, had a density of 1.7 g/cm^3^ and an average particle size of 3.5 nm, and was provided by Nanocor Inc. (Chicago, IL, USA). Nitrogen (N2), supplied by Medical Gas Beijing (Beijing, China), was used as the physical blowing agent.

### 2.2. Preparation of Compounds

Before compounding, PLA pellets were dried at 70 °C for at least 6 h to remove excess moisture. The pure PLA pellets and 5 wt % nanoclay were compounded at the temperature of 170 °C for 15 min with screw rpm of 68, using a co-rotating and intermeshing twin-screw extruder with a screw diameter of 40 mm. The melt flow rate (MFR) of prepared PLA/clay nanocomposites was 5.6 g/10 min (210 °C, 2.16 kg).

### 2.3. Experimental Setup

**Figure 1:**
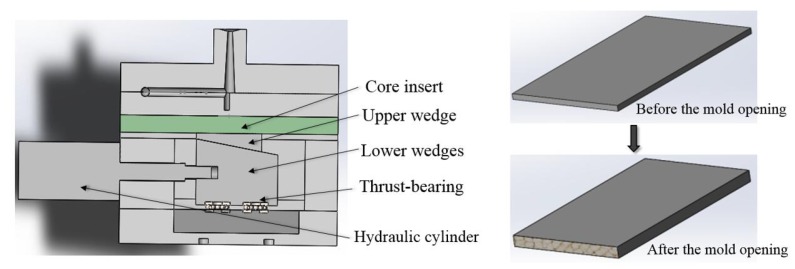
Cross-section of the mold and schematic of the procedure.

A 130 t POTENZA injection molding machine (HongKong, China) equipped Mucell gas injection unit was used to conduct the experiments. The injection machine was equipped with an opening mold. It takes about forty seconds to create excellent gas–polymer solution in the barrel before the injection. The samples with 50% fraction were fabricated using mold opening processes. Figure 1 shows a cross-section image of the mold. The core insert was moved by a wedge mechanism driven by a hydraulic cylinder. In the first injection step, the cavity thickness of 2 mm was set as a basic position for all the experiments. The cavity thickness can continuously be expanded from 2 mm to 4 mm in distance by the movable plate (Figure 1). The mold contained a rectangular cavity with a fan gate of 2 mm thickness after the sprue. The cavity dimensions were 147 × 40 × 2 mm^3^. A movable plate enables an enlargement of the cavity volume.

**Figure 2:**
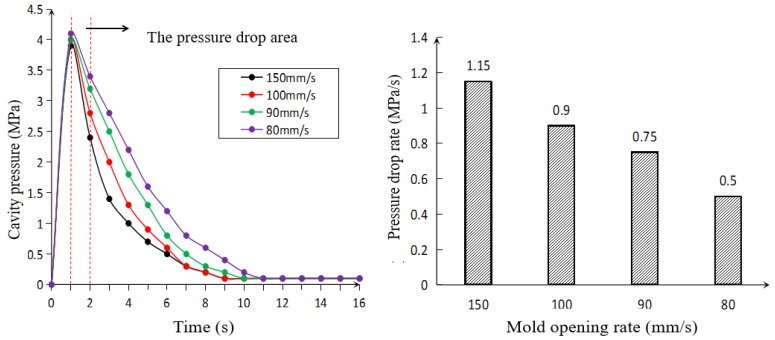
Pressure profiles at different rates of mold opening.

The measured cavity pressures during mold opening operation were shown in Figure 2. The mold cavity pressures were measured near the gate of the cavity which was a good indicator of the overall tendency for cavity pressure. As can be seen from the figure, the cavity pressure dramatically increased during the mold filling and reached a maximum at about 1 s, then the cavity expanded in the thickness direction. The pressure drop area caused by the mold opening was between 1 and 2 s, and the cavity pressure continued to decrease with the cooling of melt. The dropping rate of pressure generated in the pressure dropping area caused by the mold opening is a key parameter for foaming which has been demonstrated in previous work [16]. The chosen average values of pressure drop rate within the short time after mold opening was in accordance with the actual conditions as the mold displacement had linear change with time. The dropping rate of pressure can be regulated efficiently by controlling the rate of mold opening. Four rates of mold opening corresponding to four dropping rates of pressure (1.15, 0.9, 0.75, and 0.5 MPa/s) were studied, respectively.

The processing parameters used in the injection molding of foamed samples are summarized in Table 1. The optimized processing parameters were first obtained by a series of trial experiments [29]. This was done to acquire the preferable processing temperature for weakening the thermal degradation of PLA, which mainly depends on the melt temperature and residence time in the extruder, and to achieve relatively similar pressure profiles within the cavity [30]. The selected processing temperature was 185 °C. A melt pressure of 18 MPa was chosen to ensure that it was sufficiently higher than the solubility pressure of 9.5 MPa for the 0.6 wt % N_2_ [31].

### 2.4. Foam Characterization

To examine the cell diameter and density, the samples were prepared from the location of pressure sensor for taking SEM images, that is, near the gate of foamed samples. These samples were frozen fractured and coated with platinum using a sputter coater to enhance their conductivity. The microstructures were examined using a JSF-4700 scanning electron microscope (SEM, JEOL, Tokyo, Japan). Image processing and cell count was automatically carried out using Image-Pro Plus6 (Media Cybernetics, Inc., Rockville, MD, USA). The local cell diameter and the local cell density were calculated from the SEM micrographs according the following equation [32]:

$$cell\;diameter=\frac{\sum_{i=1}^{n}d_{i}}{n}$$

$$cell\;density={\left(\frac{nM^{2}}{A}\right)}^{\frac{3}{2}}$$

where *d_i_* is the average diameter of cell, *n* is the number of voids in the micrograph, and *M* and *A* are the magnification factor and area of the micrograph, respectively.

### 2.5. Tensile Testing

The ASTM D638-03 and ASTM D256 standards were adopted for the static tensile testing and Izod impact resistance testing of foamed samples, respectively. At least five specimens were tested for each case to decrease the random error and the average were calculated as the results. The sample was cut from the injection-molded samples, parallel to the samples’ longitudinal centerline with a slight distance from the center. The tensile tests were conducted at a temperature of 24 °C with a 50 kN load. The stretching speed was set to 10 mm/min.

### 2.6. Surface Quality Characterization

The surface roughness (*R_a_*) measuring was used to characterize the surface quality of plastic parts. The 3D laser scanning microscope (LSM) (Japan OLYMPUS Company, Tokyo, Japan) was adopted to measure surface roughness (*Ra*) of foamed samples under different pressure drop rate. Five different points of plastic surface were served as measuring positions. The average of the five points was taken as the surface roughness of the measured surface.

## 3. Results and Discussion

### 3.1. Cell Structure

**Figure 3:**
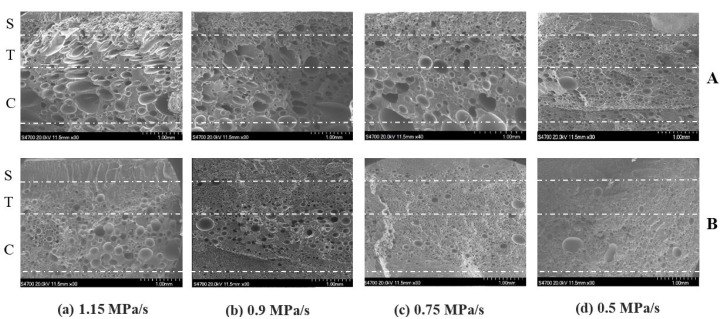
Representative SEM micrographs of the injected foams of pure PLA (**A**) and PLA–nanoclay (**B**) with four different mold opening rates. PLA, polylactide.

*Effects of mold opening rate on cell structure for pure PLA and PLA*–*nanoclay.* Figure 3 shows the representative SEM micrographs of the injected foams of pure PLA and PLA–nanoclay with four different mold opening rates. Overall, three different regions were observed in the structure across the samples’ half thickness. These regions were the skin layer, the transition region, and the core region, which were identified as S, T, and C, respectively, in Figure 3. The overall view of the cell distribution was clearly a skin–core structure without obvious interface. The skin layer presented a solid unfoamed structure, and its thickness unchanged approximately at a given injection flow rate. The cell size in the center zone is fine cells, and the coarse cells occur near the skin or in the skin close to the interface between skin and core. Before the movable plate expanded, the measured cavity pressure showed similar pressure profiles under identical processing conditions, and the cooling rate was not significantly affected by the rate of mold opening. These results indicate that different mold opening rates did not result in different thicknesses of skin layer. 

**Figure 4:**
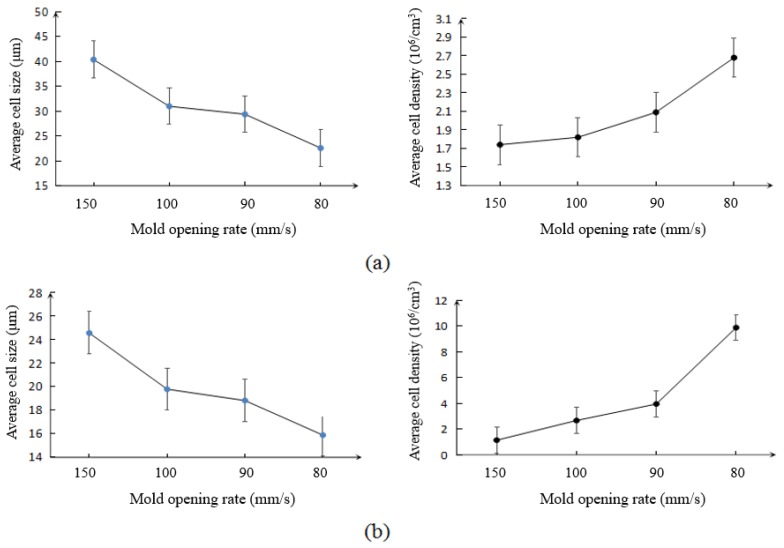
Average cell size and cell density vs. mold opening rate in (**a**) pure PLA foams and (**b**) PLA–nanoclay foams measured at the near the gate of the samples.

Both the transition and core regions possessed a cellular structure, but they had different cell morphologies. The major difference between the core and transition regions was the degree of cell elongation, which was much more severe in the core region. When decreasing the rate of mold opening, the degree of cell elongation decreased, and the cells started to distribute more uniformly in terms of size and location at both the transition and core regions. As seen in Figure 4a, the lower mold opening rate resulted in smaller cell sizes and greater cell densities. Decreasing the rate of mold opening from 150 mm/s to 80 mm/s reduced the average cell size from 40.4 μm to 22.6 μm, and increased the cell density from 1.74 × 10^6^ to 2.68 × 10^6^ cells/cm^3^, respectively. 

According to the classical theory of nucleation, the nucleation rate increases with the rate of pressure dropping. A very high pressure drop rate occurs in either the nozzle orifice or the valve gate during injection and mold filling process which results in more cell nucleation [5]. The mold opening is the secondary control of pressure drop, and the set of mold opening rate determines the pressure drop rate [24]. A large number of elongated cells were actually obtained rather than the homogeneous size of the bubble structure for the stretching force in the mold opening direction [32]. Since higher rate of pressure dropping dramatically reduced the melt pressure during mold opening and the decrease was more severe in the case of higher mold opening, thus, there was not enough melt pressure inhibition of cell nucleation and growth. The gas entered quickly into the nucleated cells within a short time in both pure PLA and PLA–nanoclay, resulting in increased pressure and tensile stress in the cell. A large amount of nucleated cells coalesced into large cells because of lower surface tension and poor cell density in the case of higher rate of mold opening. Consequently, higher rate of pressure dropping, more cell nucleation, lower melt pressure, and more severe collapse were obtained on the condition of higher rate of mold opening. On the contrary, lower rate of pressure dropping provided a circumstance where the melt had higher pressure to prevent larger number of cell nucleation from growing and coalescing within a certain range. In addition, spherical cells with greater cell density and smaller cell size occurred in the core region, where the temperature is higher than that of the transition region.

As seen in Figure 4b, the average data of the cell density and cell size of PLA–nanoclay foams have the similar trend with that of pure PLA foams. This similar trend indicated that the effect of mold opening rate on the behavior of PLA–nanoclay foaming was the same as that of pure PLA foams. The major differences between the injection foaming of PLA–nanoclay and pure PLA were in the size and density of the cells. The cell structure was changed after adding the nanoclay. In the core region, the cells were relatively smaller and more uniform in size and in distribution and retained their spherical shape during cell growth. From Figure 4b, the decrease of pressure dropping rate reduced the average cell size from 24.6 μm to 15.9 μm and increased the cell density from 1.14 × 10^6^ to 9.88 × 10^6^ cells/cm^3^. Since the addition of 5 wt % nanoclay decreased the activation energy needed for nucleation and increased the melt strength, small-sized and high-density cells were obtained easily at the same rate of mold opening [33]. A significant number of cells in the transition region also retained their spherical shape. Some big cells due to cells coalescing were observed in the core region.

### 3.2. Mechanical Properties

**Figure 5:**
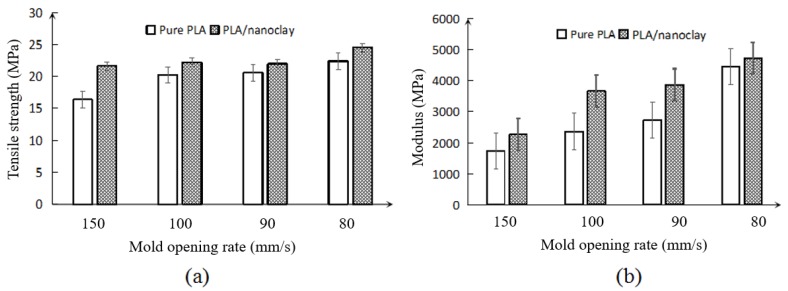
Tensile properties of foamed pure PLA and PLA–nanoclay (**a**) tensile strength; (**b**) tensile modulus.

*Effects of mold opening rate and addition of nanoclay on tensile properties*. Figure 5 shows the tensile strength and modulus for foamed pure PLA and PLA–nanoclay. For both the foamed pure PLA and PLA–nanoclay, the variation of mold opening rate changed the overall tensile strength of the PLA from a relatively brittle characteristic to a ductile one. However, with the addition of 5 wt % nanoclay during compounding, the tensile strength improved that on the as the pure PLA.

Figure 5a,b show the variations of the tensile strength and modulus of the foamed pure PLA and PLA–nanoclay samples, respectively. Overall, the tensile strength and modulus increase when decreasing the rate of mold opening. With the decreasing of rate of mold opening, the tensile strength and modulus increased. The increase in these properties was attributed to the uniformly distributed fine cells with greater cell density and smaller cell size in the PLA foams. Because of the presence of uniform smaller-sized cells, the more effective load areas of the foamed sample can be observed with lower rate of mold opening. To evaluate the effect of the added nanoclay on the tensile strength and modulus, the mechanical properties of pure PLA and PLA–nanoclay were compared. The strength of the foamed samples with the addition of 5 wt % nanoclay increased highest up to 15% than foamed pure PLA. As seen in Figure 5b, the modulus of the foamed PLA–nanoclay samples was also higher than that of the pure PLA samples, particularly at the 0.9 MPa/s and 0.75 MPa/s pressure drop rate, which was sharply higher, this phenomenon was due to the crystals induced by the nanoclay particle have acted as effective cell-nucleating agents and increased the low melt strength of PLA which resulted in improved cellular structure in PLA–nanoclay [34].

**Figure 6:**
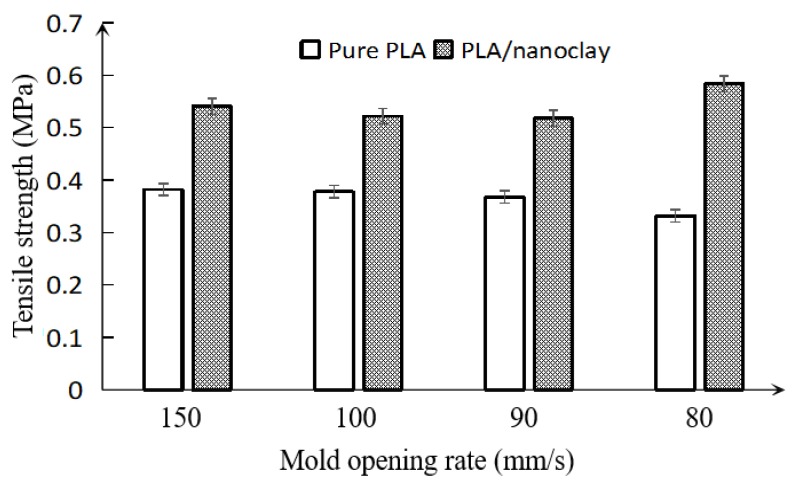
Variation of the impact resistance of pure PLA and PLA–nanoclay foamed samples with various mold opening rates.

*Effects of mold opening rate and addition of nanoclay on the impact resistance*. Figure 6 shows the Izod impact resistance of the foamed pure PLA and PLA–nanoclay samples at various pressure drop rates. Overall, with the decreasing of mold opening rate, the impact resistance of the pure PLA and PLA–nanoclay foamed samples did not significantly change, only slightly floating in their impact behavior. This phenomenon was caused by the cell size and the void fraction [35]. The smaller cell was obtained as the energy absorbers increased impact resistance when decreasing the rate of mold opening [5]. However, the impact resistance did not vary obviously which could be attributed to the slight floating of the void fraction of samples.

Compared with the pure PLA foamed samples, it was seen that the impact resistance of the foamed PLA–nanoclay samples was higher. This difference was caused by two factors. The first one is the addition of nanoclay. It enhanced crystallinity that significantly affects the impact resistance of PLA [5]. Another important factor is the cell size. The larger number of smaller-sized cells uniformly distributed in the matrix would promote the energy absorption. Even though the void fraction of PLA–nanoclay foamed samples was equal to that of pure PLA foamed samples, it had smaller-sized cells than pure PLA foamed samples. In other words, there were enough cells absorbing the impact energy in PLA–nanoclay foamed samples. Thus, the addition of nanoclay could improve the cell structure, and improve the impact resistance, which might expand the application of PLA.

Overall, the foamed samples with smaller-sized cells had higher average tensile strength and modulus compared with those of larger-sized cells. This can be attributed to the presence of certain large cells that weakened barrier effect on crack. These larger cells may become regions of stress concentrations, thereby decreasing the tensile properties of the foam. This was clearly seen in the inferior tensile properties of the pure PLA foams and PLA–nanoclay foams with larger-sized cells, and the pure PLA had poorer foam properties than the PLA–nanoclay foams. By adding nanoclay to the pure PLA, the foamed samples’ tensile properties were further improved. This was associated with the more uniform cell structure of the PLA–nanoclay foams, which reduced the number of large cells and resulted in further improved tensile properties. For the modulus, the foamed nanoclay samples had higher value than their pure PLA counterparts. The cell structure has great influence to the impact resistance and void fraction, and the material also has great impact on it.

### 3.3. Surface Quality

**Figure 7:**
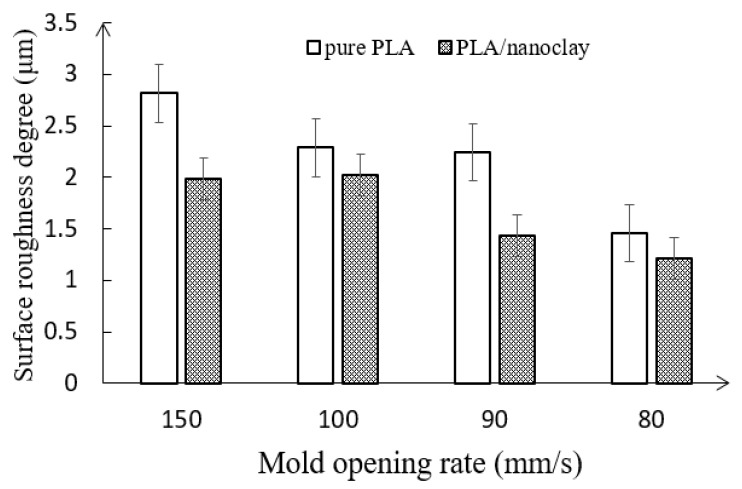
Variation of the surface quality of pure PLA and PLA–nanoclay foamed samples with various mold opening rate.

*Effects of mold opening rate and the addition of nanoclay on the surface quality*. Figure 7 shows the surface quality of foamed pure PLA and PLA–nanoclay. Bubble shearing on the interface is the major source of roughness on the surface. The cell deformation whose shape of cell gradually deforms to an oval shape increased with the distance close to the skin and moved toward the skin. The sheared cell in the surface either tears to break or stays in the oval shape below the skin [5]. There was enormous differential pressure between the melt and the mold cavity, especially between the melt surface at the time of mold expanding. The volume and surface of bubbles below the skin increased with swelling of the material, which further resulted in the strong tension stress of skin layer. It is noted that the skin layer was very thin at the moment of mold opening since the cavity expanded once the mold filling completed. A number of bubbles teared to break and escaped to the surface of the matrix to form smaller bubbles or broke holes on the surface. Thus, the spiral and crazing defect of plastic surface would appear.

As can be seen from Figure 7, the surface roughness (*R_a_*) of the microcellular parts can be effectively improved through reducing the rate of mold opening. With the decreasing of mold opening rate, the surface roughness of the foamed pure PLA samples dropped from 2.915 to 1.558 μm. Lower mold opening rate resulted in finer cells; the sheared cells with a greater cell density and smaller cell sizes that stayed below the skin were torn due to the pressure dropping between the melt surface and cavity surface, which was benefiting to form smaller bubbles on the surface [5]. In addition, the lower of pressure dropping indicated the mold opened at a slower speed. The outer surface of product and the surface of the mold core could closely contact to each other and get cooled in a short time, which was conducive to the fracture of the bubble hole on the surface of the melt and improved the surface roughness of microcellular PLA.

As mentioned previously, surface imperfections of the microcellular injection molded parts, such as swirl marks and silver streaks, were caused by bubbles forming at the melt front during the mold filling. These bubbles were transported toward the melt surface by flow behavior and were subsequently sheared and stretched against the mold wall by the flowing melt. The addition of nanoclay in the microcellular injection molding process increased the melt strength, which retarded the growth of bubbles on the surface of melt so that the probability of bubbles being formed at the melt front, and subsequently being sheared and stretched against the mold wall, dramatically decreased. It is concluded that the addition of nanoclay was beneficial to get greater surface roughness of microcellular PLA.

## 4. Conclusions

Microcellular PLA and PLA composite with void fractions as high as 50% were fabricated using microcellular injection molding with mold opening technology. The effects of mold opening rate on the foaming behavior, structural uniformity, mechanical properties, and surface quality of pure PLA and PLA–nanoclay were investigated. The effect of the addition of nanoclay to pure PLA on these properties was also identified.

PLA is challenging to be molded by ordinary foaming because of low melt strength. Compared with previous work [28], the high-pressure FIM coupled with mold opening technology can obtain void fractions as high as 50%, which was much higher than the void fractions of only 15% in the traditional HPFIM process. In addition, the mold opening injecting technology could increase the cell density and improve the overall performance of microcellular PLA. The rate of mold opening had a significant effect on cell structure, which further resulted in the variation of the PLA foams’ mechanical properties. When the rate of mold opening decreased, the average cell size also decreased, whereas the average cell density noticeably increased. These changes resulted from the difference between the inside and outside pressures of the cell. However, the rate of mold opening did not significantly affect the impact resistance of the foamed PLA. The impact resistance of the foamed PLA was associated with its void fraction.

The effect of different mold opening rates on the cell structure and mechanical properties of PLA–nanoclay foams was the same as that on pure PLA foams. The addition of 5 wt % nanoclay significantly improved the melt strength, which had a positive influence on the foaming ability and mechanical properties of PLA–nanoclay foams and achieved greater cell density and smaller cell size.

The mold opening rate also changed the PLA’s power needed for nucleation and consequently affected the surface quality. Lower rate of mold opening resulted in the finer surface roughness. The addition of nanoclay also showed significant improvement of the surface quality.

## Figures and Tables

**Table 1:** Processing parameters used in the injection molding of foamed samples.

Injection Molding Parameter	Value
Barrel temperature (°C)	185
Mold temperature (°C)	25
Screw speed (rpm)	400
Melt pressure (MPa)	18
Injecting speed (mm/s) Plasticizing stroke (mm)	100 60
N_2_ injecting pressure (MPa)	12
N_2_ wt % content (%)	0.6
Degree of mold opening (mm)	2
Mold opening delay time (s)	1
